# Consumer Preference for Rice Grain Quality in the South Kivu and Tanganyika Provinces, Eastern DR Congo

**DOI:** 10.3390/foods12213995

**Published:** 2023-10-31

**Authors:** Kilongo Bulambo, Hossein Azadi, Sylvie Polepole, Monique Nabintu, Emmanuel Bembeleza, Paul Dontsop, Jules Masimane, Barbara Haurez, Mamadou Fofana, Ludivine Lassois

**Affiliations:** 1 Plant Genetics and Rhizosphere Processes Lab, Gembloux Agro-Bio Tech, Liege University, 5030 Gembloux, Belgium; pkbulambo@student.uliege.be (K.B.); ludivine.lassois@uliege.be (L.L.); 2 Department of Economics and Rural Development, Gembloux Agro-Bio Tech, University of Liège, 5030 Gembloux, Belgium; 3 Institut International d’Agriculture Tropicale (IITA), Bukavu P.O. Box 1222, Democratic Republic of the Congo; s.polepole@cgiar.org (S.P.); kondomonic@gmail.com (M.N.); p.dontsop@cgiar.org (P.D.); julesmasimane@yahoo.fr (J.M.); ma.fofana@cgiar.org (M.F.); 4 Institut Facultaire des Sciences Agronomiques (IFA) de Yangambi, Kisangani BP 1232, Democratic Republic of the Congo; bembeleza3@gmail.com; 5 TERRA Research Centre, Gembloux Agro-Bio Tech, University of Liège, 5030 Gembloux, Belgium; barbara.haurez@uliege.be

**Keywords:** consumer attitudes, local rice, imported rice, nutritional properties, quality attributes

## Abstract

In Africa, rice has always been a staple food in some countries and the fastest growing food source there. In the Democratic Republic of Congo (DRC), in terms of cereal production, rice is ranked second after maize and is an important source of income for the rice farmer. The objective of this study was to analyze and understand consumers’ preferences and behaviors towards local and imported rice in the South Kivu and Tanganyika provinces, DRC. Data collected on 1565 rice-consuming households in eastern DRC showed that there is a great opportunity for the rice value chain and food policy development, and the promotion of local rice consumption. Consumers focus on local rice because it is cheaper, but it does not always meet their desired needs. Indeed, only urban consumers were more willing to pay for higher-quality rice. The development of the demand for local rice calls for strong investment in improving production, post-harvest practices, and market aspects. It was found that over 90% of rice consumers know about local rice production and over 84% have consumed it. In rural areas, there is typically lower consumption of imported rice. However, as households require more rice, they tend to rely more on imported varieties due to their availability in the market. The most preferred rice attributes were flavor, aroma, purity, swelling capacity, breakage rate, and whiteness. Therefore, rice producers should consider the habits and needs of consumers to improve market demand. In addition, good packaging, labeling, and marketing can also enhance local rice preference and competitiveness in South Kivu and Tanganyika provinces in eastern DRC. The findings of this study indicated that research aimed at improving local rice varieties with regard to yield, disease resistance, and organoleptic qualities could enable the population to consume more locally produced rice, which is often more affordable than imported rice. This could in turn significantly reduce the need for rice imports. These results suggest that research carried out to improve the yield and organoleptic qualities of local rice in this area can allow it to be more competitive on the market and can reduce the importation of rice.

## 1. Introduction

In the world, rice is consumed by over 3.9 billion people [1]. It is intensively cultivated in Asia, and Asians consume about 75% of the global rice supply, indicating that rice is an important food security crop in Asian countries. The estimated annual rice production in Africa was thirty-one million tons from 11 million hectares under cultivation [2]. In Africa, where the challenges of urbanization and population growth are even more pronounced than in other regions, a significant increase in rice demand is observed [3]. African producing countries import 10 to 90% of their rice needs, estimated at a cost of over USD 5.5 billion per year for the whole continent [3]. Indeed, higher urbanization is related to strong consumption preferences for imported rice because the local rice produced is not sufficient in terms of quantity and quality to meet the demand of the urban population, which is growing and too demanding in terms of rice quality [4,5]. Moreover, global demand for rice in African continues to exceed local production [6,7].

Thereby, several countries [3,8] are seeking food sovereignty by setting up food self-sufficiency policies through rice self-supply strategies. However, long-term food security cannot rely on dependency on food imports but must be built on investment in agriculture and local production [9]. Therefore, as part of global strategies, increasing local rice production using high-yielding and consumer-preferred varieties is considered one of the most sustainable and profitable options in sub-Saharan Africa [3]. Overall, it seems that there is a need for greater investment in local rice production and processing in order to improve the quality of locally produced rice and meet consumer demand. This may require changes in agricultural practices, processing techniques, and marketing strategies, as well as government support and investment. Ultimately, the goal should be to create a sustainable and thriving rice industry that can provide high-quality rice to consumers while also supporting local communities and economies [5,10,11]. 

In the Democratic Republic of Congo (DRC), rice consumption per capita per year has increased from 4.7 kg in 2010 to 7 kg in 2016 [2]. Because of the significant population and growth rate of the country, in 2017 and 2015, INS and UNICEF reported that the creation of new towns, as noted by the FAO in [12], is resulting in the development of wider consumption zones and market opportunities, respectively. These changes may influence consumer preferences, as noted by Michel et al. [7]. Therefore, it is essential for businesses to stay aware of these evolving preferences and adjust their strategies accordingly to remain competitive in the market. Hence, local rice production must be substantially increased and adapted to consumers’ demands and preferences to withstand external competition [10]. Extrinsic quality attributes like labeling, packaging, and marketing also play a powerful role in improving the reputation of local rice and its value chain, building value, and reducing quality uncertainty [7]. Once the local product is purchased, it must correspond to urban consumers’ expectations to ensure long-term consumption [11].

Regrettably, despite the DRC’s abundant land resources, prominent hydrographic network, and favorable climatic conditions for rice cultivation throughout the year, the domestic rice supply still falls short of meeting consumers’ needs in terms of both quantity and quality, as reported by the FAO [12]. In DRC, the quantity of local rice production is not enough to meet the demand of the population, and even the quality does not meet consumers’ needs [12], although the country’s land resources are abundant with favorable conditions to produce rice [9].

Projects have invested in local rice production and improvement, but the imported rice share is still larger than that of local rice [13]. African countries remain a rice importer, with more than 30% of food importation [6]. In the meantime, in some areas, rice farmers are constrained to reduce their rice cropping area in favor of other crops such as maize, sweet potato, and vegetables, following the inflow of rice importations because local rice does not meet consumers’ preferences [14,15]. The higher rice demand is a real opportunity to be seized for local rice farmers as well as for the various actors throughout the rice value chain [16]. To intensify agricultural production and improve strategic value chains in eastern DRC, the integrated project for agriculture growth in the Great Lake Region (PICAGL project) was launched in 2018 by the government. This project is implemented in the South Kivu and Tanganyika provinces. One of the principal aims of the rice value chain was to reduce its importation through the valorization of local production and the increase in its consumption [17]. In this regard, previous studies (e.g., [5,7,9,18,19]) have shown that consumers’ preferences for rice vary from one country or region to another. It is influenced by rice attributes, transformation, and cooking process [20]; by its price [21]; by labeling and marketing aspects [22]; by demographic and socio-economic factors like household income, education, age, etc. [23]; and by the purchase location and provenance [24].

The objective of this study was to analyze and understand consumers’ preferences and behaviors towards local and imported rice in the South Kivu and Tanganyika provinces, DRC, because these two provinces are very important in the production of rice in the east of the DRC; in addition, they are in the part where the PICAGL project is carried out.

Specifically, this study aimed to (i) identify the socio-economic factors influencing rice preference and (ii) analyze the appreciation for local or imported rice.

While the study addresses consumer preferences for rice grain quality in the South Kivu and Tanganyika provinces of eastern DR Congo, its implications extend far beyond this specific region. Rice, as a staple food, holds global significance, and this comprehensive analysis of 1565 rice samples offers insights into rice quality preferences that are applicable not only in Africa but also in rice-producing regions worldwide. Furthermore, the study addresses critical issues related to food security, sustainable agriculture, and cross-cultural perspectives, which have relevance on a global scale. This study can be useful for rice-producing communities around the world by influencing global discussions and policies aimed at improving rice production and quality assessment.

## 2. Materials and Methods

### 2.1. Study Sites

**Figure 1:**
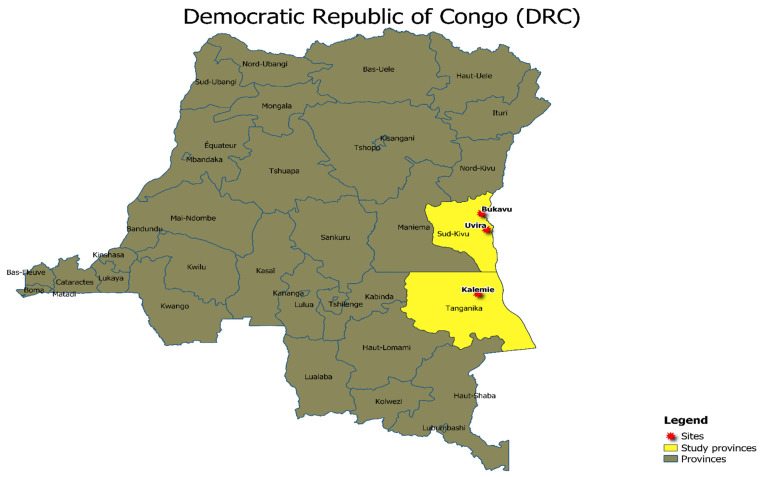
Map of the DRC with the sites used for the survey: Bukavu (2°30′34.0″ S, 28°50′49.9″ E), Uvira (3°21′32.9″ S, 29°08′25.3″ E), and Kalemie (5°54′31.2″ S, 29°11′14.8″ E) in DRC.

The study was conducted on the rice consumers of the South Kivu and Tanganyika provinces, eastern DRC; these provinces (see Figure 1) are propitious areas for rice cropping and consumption. It consisted of a survey, carried out in several areas selected from rural ones (Uvira district in South Kivu province and Kalemie district in Tanganyika province) and urban ones (Bukavu town and Uvira town in South Kivu province and Kalemie town in Tanganyika province). These areas were selected considering the rice production and consumption status, and their corresponding villages accordingly.

According to the FAO [12], rice is an important food crop in both South Kivu and Tanganyika provinces of eastern DRC. In South Kivu, rice is mainly produced in the highlands surrounding the Lake Kivu region, while in Tanganyika, it is grown in the floodplain areas of Lake Tanganyika. The majority of rice farmers in these provinces are small-scale producers who rely on traditional farming methods and have limited access to modern agricultural technologies and inputs [25]. As a result, rice yields in these regions tend to be low, and the quality of the rice produced can also be inconsistent. Despite these challenges, efforts are being made to improve rice production in these regions through various initiatives, such as the promotion of improved rice varieties, the adoption of modern farming practices, and the provision of training and support to small-scale rice farmers [26].

### 2.2. Data Collection and Analysis

Data collection for Tanganyika province was carried out from November to December 2020, and from January to February 2021 for the South Kivu province. A direct questionnaire was used to collect information using the Open Data Kit (ODK) collection tool. The questionnaire was based on information relating to rice consumption and, more specifically, the preference between local rice and imported rice, as well as the attributes of rice preferred by this population. During the survey, a total of 1565 households were selected randomly from those known to consume rice, considering a sampling interval of 20 houses in the subareas (villages). Of these, 985 households were from South Kivu province, while the remaining 580 were from Tanganyika province. The heads of these households were interviewed as part of the survey.

Data were analyzed with R software version 4.0.2 [27]. Basic descriptive statistics including means, standard error, and frequencies were computed to describe the responses with regard to the study areas. Chi-squared test and one-way analysis of variance (ANOVA) were used to examine the differences in the responses. In order to fit with linear regression assumptions for ANOVA, BoxCox power transformations were applied to the continuous variables, the ANOVA was applied to the transformed variables, and the means comparisons were applied to the back-transformed values. This study used the least squared means method and applied the least significant difference (LSD) test at a significance level of *p* < 0.05, considering appropriate error terms, to compare the means.

Generalized linear models were used for further analyses, considering the nature of the outcome variables to fit the regression analysis assumptions and avoid bias.

To gather information on the type of consumed rice (polytomous variable), a multinomial log-linear regression model was performed (Equation (1)).

(1) $$Y=\beta_{0}+\beta_{1}\;x_{1}+\cdots +\beta_{q}\;x_{q}$$

with *Y* being the multinomial dependent variable, $\beta_{1\ldots q}$ being the regression coefficients, and $x_{1\ldots q}$ being the vector of independent variables (Equation (2)), namely,

(2) $$x_{1\ldots 5}=\{Area,\;ConsFreq,\;QtyCons,\;ConsTrend,\;NbrePeopleCons\}$$

which are, respectively, the area of study, the rice consumption frequency, the quantity of consumed rice, the consumption trend, and the number of people that consume rice in the household.

To obtain information about the knowledge of local rice production and the consumption of local rice (both binary variables), a generalized linear regression with a logit link was performed, as in Equation (3):

(3) $$logit(p)=ln(\frac{p}{1-p})=\beta_{0}+\beta_{1}\;x_{1}+\cdots +\beta_{q}\;x_{q}$$

where *p* is the probability that the dependent variable equals 1/Yes, and $x_{1\ldots q}$ is the vector of explanatory variables, as in Equation (2).

An ordered regression model was applied to gather information about the confidence in the local and imported consumed rice, and about the appreciation of the local and imported consumed rice, with regards to the socio-demographic parameters (Equation (4)). The regression equation is as follows:

(4) $$Y=\beta_{0}+\beta_{1}\;x_{1}+\cdots +\beta_{p}\;x_{p}$$

where Y is the ordinal dependent variable, $\beta_{1\ldots p}$ are the regression coefficients, and $x_{1\ldots p}$ is the vector of independent variables (Equation (5)), namely,

(5) $$x_{1\ldots 10}=\{Area,BornArea,Age,Gender,Religion,MaritalSate,hhSize,School,\;Rev\;Gap,\;Income\;Source\}$$

which are, respectively, the study area, born/not born in the area, the age of the respondent, the gender, the religion, the marital status, the household size, have/have not studied at secondary school, the revenue gap, and the main source of income.

For all the considered regression models, the model parameters were estimated using the maximum likelihood method with Chi-squared test for significance, as well as relative risk ratios (odds ratio), allowing easier interpretation of the logit coefficients.

Correspondence analysis was performed on rice preference attributes, considering their mean importance rank according to the consumers’ point of view.

## 3. Results

### 3.1. Socio-Economic Characteristics of Rice Consumers

As reported in Table 1, 64% of rice consumers indicated that the village they currently reside in was their natal village, and this is greater for the people in the districts than in the towns, except Kalemie town, where 71% of people were born there.

The average age of rice consumers was 40 ± 0.7 years, 67% being women, with the highest records for Bukavu and Uvira towns (83% and 80%, respectively). This is because women are much more involved in agricultural activities, and they are the ones who are housewives.

They live in households with an average size of 8 ± 0.2 people, amongst which half are women (4 ± 0.1). Protestants and Catholics are the main religions encountered (Table 1). The high number of people in the households suggests a great opportunity for rice consumption and hence rice market and value chain development, especially with the high representation of women for dynamism in the rice value chain.

It was recorded that 78% of respondents are married, with the main source of income being agriculture and trading (36.4% and 32.3%, respectively). However, in Bukavu town, the main sources of revenue are trading (43.7%) and official work (14.3%), and in Uvira town they are trading (48.2%), agriculture (13.8%), and official work (13.0%). The monthly revenue gap is less than USD 100 for 45.6% of rice consumers and between $100 and $250for 40.2% of them (Table 1). Indeed, the amount and source of revenue have an obvious impact on the willingness to pay for rice products in quality and quantity, as consumer preference is rooted in societal norms and market behaviors [22].

### 3.2. Rice Consumption and Information on the Product

The average price of rice is USD 0.71, but our investigations showed that it is more expensive in the town of Bukavu (USD 0.80 per kg) and less expensive in the rural district of Uvira (USD 0.62 per kg) (Table 2). In fact, urban consumers are more interested in consuming rice with superior quality attributes and are prepared to pay for it [11]. The observed preference differs between rural and urban areas, with local rice more consumed in Uvira and Kalemie districts (93.8% and 76.3% of surveyed households, respectively) and imported rice more consumed in Bukavu, Uvira, and Kalemie towns (80.6%, 64.8%, and 83.4% of surveyed households, respectively) (Table 2).

Most surveyed households consume rice at least once a week (4.2% twice a day, 30.8% once a day, 35.5% three times a week, and 22.4% once a week), with only 7.3% consuming rice once a month. These figures do not significantly vary between sites. The consumed quantity is, on average, 1.7 kg per day, which is about 0.2 kg per person per day in the household. The amount of rice consumed is the highest in the Uvira rural district (1.9 kg) and the lowest in Bukavu town (1.4 kg). The rice consumption has remained stable for 41.9% of households over the past decade, while it has decreased for 33.1% of them (Table 2). Only 25% of households increase their consumption of rice; hence, agriculture needs to be supported and the rice value chain must be seriously improved [11].

This suggests advertising and sensitization consideration, as labeling is an important part of the market and communication between producers and consumers [21].

They are somewhat confident in local rice products (52.6%) and highly appreciate them (70.4%), with the same trend for imported rice (with 41.8 being somewhat confident and 60.3% having high appreciation). Demont and Ndour [11] and Zhou et al. [22] have shown that improving the quality of local rice will make it competitive in the market.

### 3.3. Appreciation of the Consumed Rice

The study on appreciation towards the local and imported rice (Table 3) showed that, compared with the city of Bukavu, imported rice was more popular in the rural district of Uvira and the towns of Uvira and Kalemie. Female gender also had a positive influence (0.6) on the appreciation of imported rice. We also observed that religion seems to have an influence on the preference for local rice, such as being Catholic compared to being Protestant. However, belonging to the New Apostolic religion or to another religion had a positive influence on the appreciation of local rice. On the other hand, being Neo-apostle or people from another religion has a positive influence on the appreciation of local rice. Separated people appreciate local rice much more than married households, while widowed people tend to appreciate local rice less than married households. Hence, this study confirms that social status impacts rice preference and consumption, as previously observed [28,29].

Households led by an educated head are twice as likely to prioritize local rice and are 40% less likely to favor imported rice. The sources of revenue art/construction, trading/business, official work, and no work/none negatively influence the appreciation of local rice compared to agricultural households (by −1.7, −1.0, −1.5, and −2.0, respectively, for an increase of one unit in the respective variable scores, keeping all others constant). It is 1.8 times more likely to prefer imported rice when the household has official work as the main revenue source, compared to those with agriculture as the main revenue (Table 4). This shows that to increase the consumption of local rice, the status and habits of the consumer should be considered [5,7,21].

### 3.4. Rice Attribute Preference

**Figure 2:**
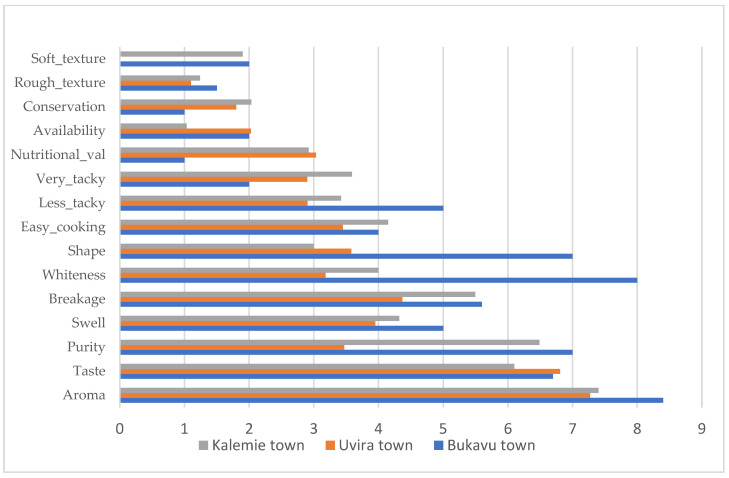
Preference towards rice attributes in Kalemie town, Uvira town, and Bukavu town.

**Figure 3:**
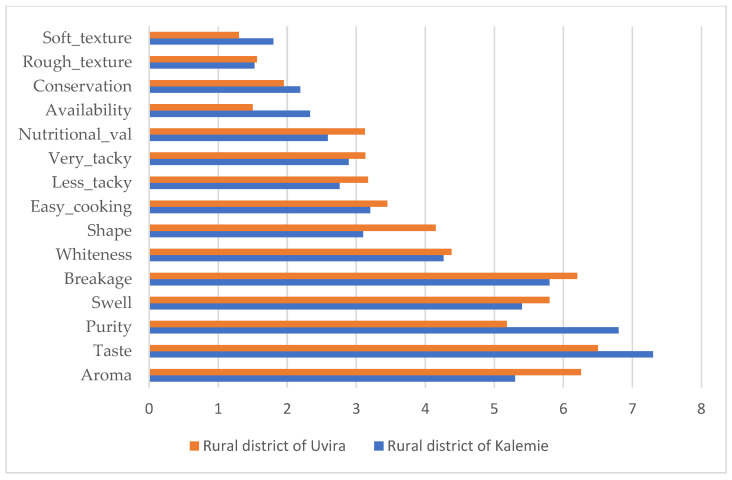
Preference towards rice attributes in rural district of Uvira and rural district of Kalemie.

**Figure 4:**
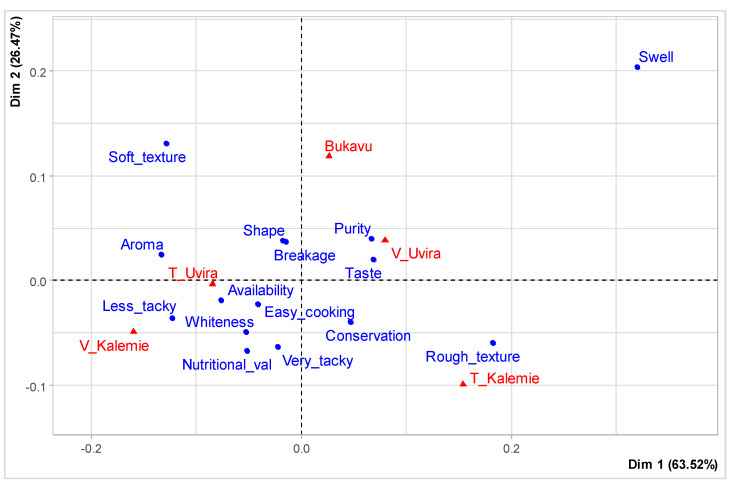
Correspondence analysis of the preference towards rice attributes in Bukavu, Uvira town, rural district of Uvira, Kalemie town, and rural district of Kalemie. The words in blue show the preferred attributes of the rice and the words in red show the names of the sites. The symbol V = Town and the symbol T = Territory. The triangle shows the sites, and the dot shows the preferred attributes of the rice.

The physical appearance of rice is among the most important attributes for rice appreciation in the market. Taste, aroma, purity, swelling, breakage rate, and whiteness were ranked as the main preferred rice attributes (Figure 2 and Figure 3). Results of the correspondence analysis, as shown in Figure 4, considering two dimensions, explained 90% of inertia. It shows that Uvira town consumers pay more attention to attributes such as the aroma, taste, and purity of rice; those from Kalemie town, Kalemie district, and Uvira rural district focus on attributes such as aroma, taste, purity, and swelling of rice; and the consumers mainly consider attributes such as aroma, taste, purity, and swelling of rice. Our results on rice attribute preferences in the study area confirm the results of Asante et al. [21] and Motbainor and Taye [23], who showed that the majority of consumers prefer rice with good attributes, namely taste, cooking quality, and aroma. According to Britwum and Demont [11], the particular ability of rice to swell upon cooking is an important quality trait for most consumers in developing countries. This is in accordance with our findings. However, the request for quality rice with good attributes has to go with good packaging and labeling [21,30].

One of the limitations of this study is that it only focuses on two provinces of eastern DR Congo, South Kivu and Tanganyika. While these regions are significant producers and consumers of rice, they may not necessarily be representative of other regions in the country, which may have different consumer preferences and market dynamics. Additionally, the study relied on self-reported data from household heads, which may be subject to recall bias and may not reflect the preferences of other household members. Finally, the study did not investigate the impact of socio-economic factors such as income and education on consumer preferences, which may play an important role in shaping these preferences.

This study on consumer preferences for rice grain quality in South Kivu and Tanganyika provinces, eastern DR Congo, adds to global knowledge in several ways. First, it contributes to our understanding of the factors that influence consumer preferences for rice quality in developing countries, where rice is a staple food and local producers compete with imported products. Second, the study highlights the importance of factors such as taste, aroma, texture, and appearance in shaping consumer preferences, which can inform the development of effective marketing strategies for rice producers both in DR Congo and in other countries. Finally, the study underscores the importance of investing in research and development to improve the quality of locally produced rice and ensuring that rice producers have access to the information and resources they need to meet consumer demand and compete effectively in the global market. Overall, the study’s findings have implications for the global rice industry and can contribute to efforts to promote sustainable and equitable development in rice-producing regions around the world.

This study holds substantial implications for both the local rice industry in South Kivu and Tanganyika provinces and the broader context of developing countries. The preference for imported rice in rice-producing regions underscores the urgency of addressing issues related to local rice quality and labeling. Clear and accurate labeling, as well as an emphasis on enhancing the attributes most preferred by consumers, such as taste, aroma, and cleanliness, can strengthen the competitiveness of local rice. Moreover, the study emphasizes the importance of recognizing consumer status, habits, and preferences, which can guide local rice producers in tailoring their products to meet specific demands. Beyond the immediate implications for local producers, this study contributes to the theoretical understanding of consumer behavior in developing nations, revealing the complex interplay between socio-economic factors and food preferences. To support sustainable rice production and development, future research directions should have more focus on socio-economic determinants, explore innovative marketing strategies, and prioritize quality enhancement in rice production processes. This can pave the way for more comprehensive initiatives that not only support the local rice industry in the provinces of South Kivu and Tanganyika but also serve as a model for other regions facing similar issues in enhancing local food production and consumption.

## 4. Conclusions

The aim of this study was to investigate consumer preferences and behaviors towards local and imported rice in South Kivu and Tanganyika provinces in the Democratic Republic of Congo. The findings of the study showed that consumers in rice-producing areas rely more on imported rice, which is supplied without proper labeling, due to concerns about the quality and quantity of domestically produced rice. To enhance the competitiveness of local rice, it is crucial for producers to ensure clear and accurate labeling. Higher-quality rice is particularly in demand in urban markets, emphasizing the significance of both rice characteristics and packaging. Therefore, producers should align their products with consumer needs and preferences, focusing on attributes like flavor, aroma, cleanliness, swelling capacity, breakage rate, and whiteness. Additionally, recognizing consumers’ status, habits, and requirements is essential. The findings of this study not only inform the local rice industry in South Kivu and Tanganyika but also offer theoretical implications for understanding consumer behavior in developing countries. It underscores the need for research and development in improving locally produced rice quality and empowering producers to meet consumer demands effectively. Future research directions could explore socio-economic factors, marketing strategies, and quality improvement to support sustainable development in rice-producing regions.

This study provides valuable insights into consumer preferences for rice quality in South Kivu and Tanganyika provinces, shedding light on factors influencing consumer behavior in developing countries. It has theoretical implications for understanding food product preferences and practical applications for local rice producers and policymakers. Future research avenues could delve deeper into socio-economic factors, marketing strategies, and quality enhancement to further support sustainable rice production and development in similar regions.

## Figures and Tables

**Table 1:** Socioeconomic characteristics of the respondents.

Variables	Area						
	South Kivu			Tanganyika			
	Rural District	Town		Rural District	Town	Total (N = 1565)	Statistic χ^2^ or *F* Value
	Uvira Rural District (n = 242)	Bukavu Town (n = 490)	Uvira Town (n = 253)	Kalemie Rural District (n = 93)	Kalemie Town (n = 487)		
Born in the area (%)	75.21	57.76	52.96	61.29	71.25	63.69	46.32 ***
Age	39.54 ^b^	37.23 ^b^	36.94 ^b^	40.76 ^b^	45.23 ^a^	40.24	24.26 ***
Gender (%)							125.41 ***
Female	51.24	83.27	79.45	63.44	57.49	66.98	
Male	48.76	16.73	20.55	36.56	42.51	33.02	
Household size	7.36 ^b^	7.92 ^ab^	7.61 ^ab^	7.18 ^b^	8.12 ^a^	7.80	3.13 *
Number of women in the household	4.18	4.22	4.18	3.71	4.32	4.21	1.38 ns
Religion (%)							111.21 ***
Catholic	30.58	50.61	28.46	29.03	30.60	33.86	
Muslim	2.07	1.84	9.49	2.15	6.57	4.42	
Neo-Apostle	0.41	0.00	1.19	0.00	2.05	0.73	
Protestant	60.74	44.08	55.73	63.44	53.39	55.48	
Jehovah Witness	1.65	1.63	1.58	2.15	1.44	1.69	
Marital status (%)							115.36 ***
Single	11.57	23.88	18.97	5.38	4.11	12.78	
Divorced	4.13	2.04	1.98	0.00	1.23	1.88	
Married	78.10	66.33	71.94	89.25	84.39	78.00	
Separated	1.24	0.00	0.00	0.00	0.62	0.37	
Widow	4.96	7.76	7.11	5.38	9.65	6.97	
Other	2.48	1.43	2.77	3.23	3.29	2.64	
None	2.07	0.41	0.79	0.00	2.67	1.19	
Revenue source (%)							594.90 ***
Agriculture	78.93	2.45	13.83	61.29	25.67	36.43	
Art/Construction	2.48	10.00	8.30	0.00	8.62	5.88	
Trading	13.22	43.67	48.22	22.58	33.88	32.31	
Teaching	1.65	7.76	1.98	4.30	5.54	4.25	
Official	1.65	14.29	13.04	7.53	10.06	9.31	
None	0.00	11.63	4.35	1.08	6.98	4.81	
Other	2.07	10.20	10.28	3.23	9.24	7.00	
Revenue gap (%)							51.65 ***
<USD 100	40.50	41.02	46.25	55.91	44.35	45.61	
>=USD 1000	0.83	1.43	0.40	3.23	5.13	2.20	
USD 100 to <250	47.11	38.98	40.71	36.56	37.58	40.19	
USD 250 to <500	11.16	15.71	11.46	4.30	11.09	10.74	
USD 500 to <1000	0.41	2.86	1.19	0.00	1.85	1.26	

Numeric variables are presented as mean value and standard error. * and *** indicate statistical significance, respectively, at the 0.05 and 0.001 levels; ns: no significant difference. a, b, c = Means followed by same letter are not significantly (*p* ≤ 0.05) different.

**Table 2:** Rice consumption in the household and information in the product.

Variables	Area						
	South Kivu			Tanganyika			
	Rural District	Town		Rural District	Town	Total (N = 1565)	Statistic χ^2^ or *F* Value
	Uvira Rural District (n = 242)	Bukavu Town (n = 490)	Uvira Town (n = 253)	Kalemie Rural District (n = 93)	Kalemie Town (n = 487)		
1 kg rice average price (USD)	0.62 ^c^	0.80 ^a^	0.68 ^b^	0.65 ^bc^	0.66 ^bc^	0.71	82.35 ***
Imported	0.74 ^ab^	0.80 ^a^	0.68 ^c^	0.82 ^a^	0.66 ^c^	0.73	46.68 ***
Local	0.62 ^b^	0.81 ^a^	0.65 ^b^	0.59 ^b^	0.69 ^ab^	0.65	19.05 ***
Type of consumed rice (%)							879.50 ***
Imported	1.65	80.61	64.82	13.98	83.37	48.89	
Both	4.55	10.41	17	9.68	9.65	10.26	
Local	93.8	8.98	18.18	76.34	6.98	40.86	
Consumption frequency (%)							85.8 ***
Twice/day	6.20	4.29	6.72	1.08	2.46	4.15	
Three times/week	40.08	30.00	31.23	38.71	37.37	35.48	
Once/day	25.62	42.45	37.94	24.73	23.00	30.75	
Once/week	23.55	17.14	19.37	26.88	24.85	22.36	
Once/month	4.55	6.12	4.74	8.60	12.32	7.27	
Consumption quantity (Kg/day)	1.93 ^a^	1.42 ^c^	1.80 ^ab^	1.72 ^ab^	1.70 ^b^	1.67	14.73 ***
Consumption trend (%)							29.55 ***
Increasing	26.45	23.88	20.16	29.03	25.67	25.04	
Static	40.91	48.98	51.78	30.11	37.58	41.87	
Decreasing	32.64	27.14	28.06	40.86	36.76	33.09	
Knowledge of local rice production (%)	100.00	81.93	99.39	92.31	85.33	91.79	32.05 ***
Have already consumed local rice (%)	100.00	62.38	93.29	92.31	73.35	84.27	61.02 ***

Numeric variables are presented as mean value and standard error. *** indicates statistical significance at the 0.001 levels. a, b, c = Means followed by same letter are not significantly (*p* ≤ 0.05) different.

**Table 3:** Type of consumed rice, knowledge of local rice, and consumption.

Variable/Parameter	Dependent Variable											
	Consumed Imported			Consumed Local			Knowledge Local Prod.			Already Consumed Locally		
	Estimate	OR	SE	Estim.	OR	SE	Estimate	OR	SE	Estimate	OR	SE
Uvira Town	−0.76 ***	0.47 ***	0.27	0.15	1.16	0.34	3.44 ***	31.2 ***	1.02	2.05 ***	7.73 ***	0.35
Bukavu Town	0.11	1.13	0.26	−0.21	1.38	0.40	2.89	28.2	1.08	1.99	5.08	0.20
Uvira Rural District	−3.00 ***	0.05 ***	0.62	3.09 ***	22.0 ***	0.41	14.11	13.36	0.39	14.10	13.36	0.28
Kalemie Town	0.13	1.14	0.25	−0.29	0.75	0.34	0.51 *	1.67 *	0.22	0.57 **	1.77 **	0.18
Kalemie Rural District	−1.63 ***	0.20 ***	0.49	2.20 ***	9.01 ***	0.45	1.07	2.90	1.07	1.95	7.04	1.06
Consumed twice a day	−0.08	0.92	0.52	0.47	1.59	0.62	0.33	1.39	0.77	0.07	1.08	0.47
Consumed three times a week	−0.25	0.78	0.24	0.18	1.20	0.30	−0.22	0.80	0.28	0.00	0.10	0.20
Consumed once a month	−0.01	0.99	0.45	0.40	1.50	0.54	−1.51 ***	0.22 ***	0.35	−0.80 **	0.45 **	0.30
Consumed once a week	−0.63 **	0.53 **	0.27	0.12	1.12	0.32	−0.74 *	0.48 *	0.30	−0.08	0.93	0.24
Consumed quantity	−0.06	0.94	0.11	−0.059	0.95	0.14	0.19	1.21	0.14	0.18	1.19	0.11
Cons. trend increasing	−0.70 ***	0.50 ***	0.24	−0.54 *	0.58 *	0.30	−0.22	0.80	0.28	−0.23	0.80	0.21
Cons. trend decreasing	−0.24	0.79	0.24	−0.24	0.79	0.28	−0.09	0.92	0.24	0.10	1.10	0.19
Nbr people consuming	−0.01	1.00	0.03	0.02	1.02	0.04	0.08 *	1.09 *	0.04	0.07 *	1.07 *	0.03
Constant	2.78 ***	16.1 ***	0.33	0.02	1.02	0.42	0.95 **	2.60 **	0.34	−0.18	0.83	0.26
Loglikelihood							−304.59			−452.43		
Akaike Inf. Crit.	1621.67						635.18			930.86		

*, **, and *** indicate statistical significance, respectively, at 0.05, 0.01, and 0.001 levels. SE: standard error of the difference of means; OR: odds ratio.

**Table 4:** Confidence and appreciation towards consumed local and imported rice.

Variable/Parameter	Dependent Variable											
	Confidence Local			Confidence Imported			Appreciation Local			Appreciation Imported		
	Estimate	OR	SE	Estimate	OR	SE	Estimate	OR	SE.	Estimate	OR	SE.
Uvira Town	−1.21 *	0.30 *	0.49	0.88 ***	2.41 ***	0.18	−1.34 *	0.26 *	0.57	2.40 ***	10.99 ***	0.24
Bukavu Town	−1.25	0.60	0.38	0.40	1.88	0.54	−0.49	0.50	0.28	2.67	3.43	0.32
Uvira Rural District	−0.92 *	0.40 *	0.45	1.46	4.29	0.82	−0.51	0.60	0.54	15.30 ***	4.4 × 10^6^ ***	0.00
Kalemie Town	−0.06	0.94	0.55	0.32 *	1.38 *	0.16	0.00	0.10	0.68	0.39 *	1.47 *	0.16
Kalemie Rural District	−0.21	0.81	0.50	0.98	2.66	0.66	−0.71	0.49	0.58	0.99	2.68	0.54
Born in area: Yes	−0.11	0.89	0.24	−0.06	0.94	0.13	−0.42	0.66	0.29	−0.07	0.94	0.14
Age	0.01	1.01	0.01	0.01	1.01	0.01	0.03	1.03	0.01	0.00	0.10	0.01
Gender: Female	−0.27	0.76	0.24	0.46 **	1.59 **	0.16	0.30	1.35	0.28	0.57 ***	1.77 ***	0.16
Religion: None	−0.86	0.42	0.86	0.056	1.06	0.49	−0.05	0.95	0.92	0.77	2.16	0.55
Religion: Other	0.58	1.795	0.60	−0.69	0.50	0.38	18.03 ***	6.8 × 10^7^ ***	0.00	−0.05	0.96	0.44
Religion: Catholic	−0.14	0.87	0.24	−0.15	0.87	0.14	−0.54 *	0.58 *	0.27	−0.08	0.95	0.14
Religion: Muslim	−0.13	0.88	0.58	0.36	1.47	0.29	0.46	1.59	0.76	0.17	1.18	0,31
Religion: Neo-Apostle	−1.42	0.24	1.99	−0.17	0.84	0.58	18.17 ***	7.8 × 10^7^ ***	0.00	0.25	1.29	0.67
Religion: Jehovah	−0.53	0.59	0.81	0.89	2.43	0.55	−0.34	0.71	0.92	0.64	1.89	0.57
Marital status: Single	0.47	1.61	0.37	0.02	1.02	0.19	0.55	1.73	0.43	0.03	1.03	0.21
Marital status: Divorced	0.08	1.08	0.64	−0.55	0.58	0.49	1.20	3.31	1.12	−0.49	0.62	0.52
Marital status: Separated	2.13	8.44	1.25	−0.59	0.56	1.03	17.21 ***	2.9 × 10^7^ ***	0.00	−1.21	0.30	1.02
Marital status: Widow	−0.50	0.61	0.50	−0.20	0.82	0.25	−1.38 *	0.25 *	0.59	−0.01	0.99	0.26
HH size	−0.01	0.99	0.03	−0.02	0.98	0.02	−0.05	0.96	0.04	−0.03	0.97	0.02
Schooling: Yes	−0.46	0.63	0.29	−0.30	0.74	0.21	0.68 *	1.97 *	0.33	−0.45 *	0.64 *	0.21
Revenue >= USD 1000	−0.49	0.62	1.51	−0.60	0.55	0.37	−1.51	0.22	1.72	−0.30	0.75	0.36
Revenue USD 100 to <250	0.91 ***	2.49 ***	0.23	0.04	1.04	0.14	0.32	1.38	0.27	0.22	1.25	0.15
Revenue USD 250 to <500	0.63	1.88	0.40	0.22	1.24	0.19	−0.07	0.93	0.47	0.17	1.19	0.21
Revenue USD 500 to <1000	−1.34	0.26	1.51	0.49	1.63	0.45	−0.39	0.68	1.53	0.90	2.47	0.49
Revenue source: None	−1.26	0.28	0.84	0.06	1.06	0.28	−2.05 *	0.13 *	0.83	0.13	1.14	0.29
Revenue source: Art/Construct	−1.39 *	0.25 *	0.63	0.43	1.54	0.28	−1.68 **	0.19 **	0.61	0.19	1.20	0.29
Revenue source: Other	−0.68	0.51	0.53	0.50	1.65	0.27	−0.48	0.62	0.59	0.35	1.41	0.28
Revenue source: Trading	−0.26	0.77	0.31	0.57 ***	1.78 ***	0.21	−1.00 **	0.37 **	0.34	0.27	1.31	0.22
Revenue source: Teaching	−0.14	0.87	0.68	0.48	1.61	0.31	−0.67	0.51	0.89	0.40	1.49	0.32
Revenue source: Official	−1.11	0.33	0.57	0.48	1.61	0.26	−1.52 *	0.22 *	0.59	0.59 *	1.81 *	0.27
AIC	804.5			2613.4			550.3			2179.0		
Loglikelihood	−369.3			−1109.0			−242.2			−1056.5		

*, **, and *** indicate statistical significance, respectively, at the 0.05, 0.01, and 0.001 levels. Estim.: estimate; SE: standard error of the difference of means; OR: odds ratio.

## Data Availability

The data that support the findings of this study are available from the authors upon reasonable request.
